# A New Theory of the Action of Mercury

**Published:** 1880-07

**Authors:** 


### Selections.

### A NEW THEORY OF THE ACTION OF MERCURY.

Dr. S. V. Clevenger, in the *Chicago Medical Gazette*, proposes a new theory of the action of mercury.

The *modus operandi* of mercury, in common with that of many other drugs whose effects are so manifest and direct, has heretofore received no satisfactory explanation. The theories advanced have been largely mere conjectures. Dr. Clevenger's theory is that mercury acts purely mechanically, and the experiments he records in support of his views certainly seem to corroborate these views. That metallic mercury applied externally may enter the circulation, is not doubted by the physiologist, the demonstration of the possibility being a standard physiological experiment. Dr. Clevenger's theory comes in conflict with that usually entertained (if it may be said that any theory is generally accepted) in the dealing with salts of mercury. He maintains that these salts are reduced either before absorption or soon after, and that as salts they do not circulate, their being no recombination after the pimary decomposition. The metallic mercury in the blood is carried unchanged into the glandular tubules, and forces its way to their blind extremities, and by their superior weight displacing the occupants of these tubules, be they normal or morbid matter. In this manner mercury acts as a deobstruent on the same principle as that in which cannon balls dropped into a pipe removes matter of lesser specific gravity. In the intestines the increased peristalsis excited by the foreign substances facilitates the progress of the minute globules, and their reaching the hepatic parenchyma. The presence of the mercury in the salivary glands stimulates their secretion, and being a foreign substance seeking for egress, sets up the changes characteristic of mercurial salivation.

" Mercurials load the circulation and emunctories with effete matter because of their deobstruent effects and ability to insinuate their particle among all tissues, separating the morbid or ulcerated portions from the healthy, by the great and universal law of heavy bodies acting in the line of 'least resistance.' If the bile is improperly diverted or suppressed, it restores it, by opening the channels through which it normally flows; if superabundant from organic obstruction, it would regulate its quantity in the same way by affording exit for morbific causes. Its aplastic action is ascribable to the capillary and lymphatic cleansing its passing would produce; the million minute globules pushing open circulatory channels and preventing accumulation, as well as affording means for absorption. Provisional callous and wound-healing would be interfered with by globules breaking up new tissue and interfering with its formation, as would any foreign substance. Mercury has been retorted over in considerable quantities from the bones of those who have died from mercurial cachexia, the little particles finding stopping places in the cancellated tissue removed from more active circulatory influences, and, in excess, doubtless dissecting away the periosteum, filling the lacunæ and canaliculi, thus unavoidably producing caries.

"The occasional tonic influence of the metal would follow wherever glandular obstruction was superinducing diminution of the red blood corpuscles, as insomnia may be overcome by bromides removing the cause, while no one assigns the bromides a place among hypnotics.

"Mercury is not a tonic; but if it increases secretion, removes obstructions and sets the corpuscular manufactories in order, as it does the biliary, it induces tonicity as the bromides induce sleep.

"But mercury also causes anæmia, which might be expected by persisting in its use, remembering its occlusive power in closing the minute passages and tubular structures which, in medicinal quantities, removed pre-existing obstructions.

"Mercury in larger doses diminishes the number of red blood corpuscles and produces anæmia, emaciation, ulceration, febrile symptoms, with a peculiar 'jerking, thready' pulse. Obviously an effect which might be salutary upon the glandular system, wrought by small doses, could become pernicious by overdoses, and hæmatosis be seriously interfered with by the vascular stasis induced by mercurial plugging of the arterioles and venioles. Any irritation causing perversion of the hepatic and splenic functions, certainly could only be followed by hæmic degeneration, and I am inclined to think that the pulse characterizing hydrargyria is due to the irregular but frequent propulsion of blood by *vis a tergo* clearing of the lesser vessels, where the metallic globules had for a while backed up the current until forcibly overcome. This brings us to the consideration of the nervous phenomena among its toxic effects.

"As to the so-called 'specific' reputation of mercury in syphilis treatment and its modus operandi, I might be excused detailing probabilities until the pathology of the complaint is better understood. The disposition of the virus being to centralize itself upon and destroy certain areas, it seems likely that the metal may, by attacking such weakened points, not only break them down, but prevent the static degeneration necessary for ulcerative processes. This, with the antagonism the metal has for occlusion anywhere, except what it induces itself in great doses, would suffice as a tentative view until we demonstrate exactly the cause of both the disease and its cure.

"The incendiary can do no harm to society while the police are alert and keep him 'moving on.' Syphilis, though in the blood, may not manifest itself if sufficient globules are chasing it from forming nuclei; but where the fluids of the body are saturated with syphiliting points enough to produce tertiary symptoms, how futile must any attempt be to restore health by any doses of the drug under consideration. The disease itself is depleting the system at this stage, and mercury but adds to the trouble, having more carious and degenerated spots to work upon.

"In short, at this period both syphilis and mercury will fraternize against the body as against a common enemy. Tonics might arrest the cachexia induced by either or both, and in addition the iodides which are known to act upon this disease and its putative cure should be given."

Dr. Clevenger's views have the advantage of being definite, at least, and their plausibility will, we think, be very generally conceded, although they may fail to satisfy. They are certainly noteworthy, and have in them the possibility of a revolution in the therapeutics of many diseases for which mercury and its salts have been empirically and blindly administered.—*Michigan Med. News.*

